# Deficits in Acoustic Startle Reactivity and Auditory Temporal Processing after Traumatic Brain Injury

**DOI:** 10.1089/neur.2021.0077

**Published:** 2022-05-06

**Authors:** Steven W. Threlkeld, Emma Morales Cestero, John Marshall, Joanna Szmydynger-Chodobska, Adam Chodobski

**Affiliations:** ^1^ Department of Neuroscience, Regis College, Weston, Massachusetts, USA.; ^2^ Department of Molecular Pharmacology, Physiology, and Biotechnology, Department of Emergency Medicine, Alpert Medical School, Brown University, Providence, Rhode Island, USA.; ^3^ Neurotrauma and Brain Barriers Research Laboratory, Department of Emergency Medicine, Alpert Medical School, Brown University, Providence, Rhode Island, USA.

**Keywords:** central auditory processing deficits, modified acoustic startle, traumatic brain injury

## Abstract

Traumatic brain injury (TBI) exacts significant neurological and financial costs on patients and their families. In adult patients with moderate-to-severe TBI, central auditory impairments have been reported. These auditory impairments may interfere with language receptivity, as observed in children with developmental brain injury. Although rodent models of TBI have been widely used to examine behavioral outcomes, few studies have evaluated how TBI affects higher-order central auditory processing across a range of cue complexities. Here, auditory processing was assessed using a modified acoustic startle paradigm. We used a battery of progressively complex stimuli (single-tone, silent gaps in white noise, and frequency-modulated [FM] sweeps) in adult rats that received unilateral controlled cortical impact injury. TBI subjects showed significant reductions in acoustic startle absolute responses across nearly all stimuli, regardless of cue, duration of stimuli, or cue complexity. Despite this overall reduction of startle magnitudes in injured animals, the detection of single-tone stimuli was comparable between TBI and sham-injured subjects, indicating intact hearing after TBI. TBI subjects showed deficits in rapid gap (5 ms) and FM sweep (175 ms) detection, and, in contrast to shams, they did not improve on detecting silent gaps and FM sweeps across days of testing. Our findings provide evidence for both low-level (brainstem-mediated) and higher-order central auditory processing deficits in a rodent model of TBI, which parallel sensory impairments observed in TBI patients. The present findings support the use of modified pre-pule auditory detection paradigms to investigate clinically relevant processes in TBI.

## Introduction

Moderate-to-severe traumatic brain injury (TBI) constitutes 10–30% of an estimated 1.7 million cases of TBI reported annually in the United States.^1^ TBI results in a complex amalgam of impairments in auditory processing and learning, which disturb an array of typical life functions, exerting significant personal and economic cost.^2^ TBI is increasingly recognized as a disease process lasting weeks, months, or years after an accident rather than an acute singular event.^3^ With this in mind, cognitive and sensory outcomes have been reported to vary depending on the timing of post-injury assessment and task demand.^4^ Given the complexity of factors associated with injury progression and limitations in measuring neurobiological processes in TBI patients, animal models continue to be a vital resource for investigating multi-domain profiles of impairment that could be used as post-injury assessment targets for treatment efficacy.^5^

Deficits in auditory temporal processing have been reported in rodent models of developmental brain injury (neonatal hypoxia-ischemia, microgyria, and cortical heterotopia)^6,7^ and after TBI in adult rats.^8^ The fact that the auditory processing deficits are observed across diverse brain injuries, with insults occurring at different ages, suggests that these processes are highly sensitive to disruption. Therefore, they may represent useful targets for pre-clinical assessment. Clinical studies show cortically mediated auditory and orienting deficits in patients with moderate-to-severe TBI.^9–12^ Specifically, event-related potential recordings in TBI patients who presented with complex tone and frequency discrimination tasks indicate disruption in cortical responses and sensory discrimination.^9–11^ In addition, deficits in auditory processing have been reported to predict language-learning outcomes in infants with developmental brain injury.^13–15^ Reduced acoustic startle magnitude has also been reported in TBI patients and rodent models.^16^ Given these reports of auditory processing deficits resulting from TBI, we investigated this phenomenon in rats using a modified acoustic startle paradigm, which involved a battery of progressively complex stimuli (single-tone, silent-gaps in white noise, and frequency-modulated [FM] sweeps).

## Methods

### Animal assurance

**FIG. 1.**
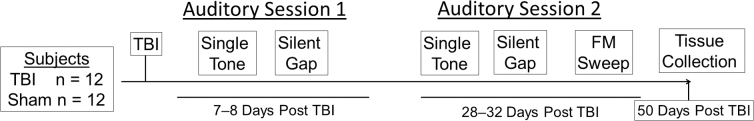
Diagram outlining the study timeline, including the number of subjects, auditory testing sequence, and the study end-point (tissue collection). FM, frequency-modulated; TBI, traumatic brain injury.

Surgical procedures were performed at Rhode Island Hospital (RIH). After injury, animals were allowed to recover for 48 h before transport to Regis College for assessments. Once at Regis College, animals were given ∼5 days to acclimate before initiation of behavioral testing (see Fig. 1 for study timeline). Surgical, animal care, and transfer procedures used in these studies were approved by the Institutional Animal Care and Use Committee (IACUC) of RIH. Behavioral testing protocols, animal care, transport procedures, and methods of euthanasia were approved by the Regis College IACUC. All procedures followed the American Veterinary Medical Association guidelines and the U.S. Department of Agriculture Guide for the Care and Use of Laboratory Animals. All procedures conformed to international guidelines on the ethical use of animals for research.

### Animals

Twenty-four adult male Long-Evans rats (TBI, *n* = 12; sham, *n* = 12), weighing 250–275 g, were purchased from the Harlan Breeding Laboratory (Indianapolis, IN). Animals were kept in the RIH animal housing facility at 22°C, with a 12-h light/dark cycle, and were maintained on standard rat food and water *ad libitum*. Animals were 8–9 weeks of age at the time of injury.

### Brain injury procedures

The controlled cortical impact (CCI) model of TBI was used as previously described.^8^ Before surgery, rats were randomly selected for inclusion into one of two groups: TBI or sham-injury. In brief, rats were anesthetized (intraperitoneal pentobarbital sodium, 60 mg/kg) and a 4-mm craniotomy was performed on the right side of the skull to expose the dura, with the center of the opening located 3.0 mm posterior to bregma and 2.5 mm lateral to the midline. Injury was produced using a CCI device purchased from Michalowski Inc. (Zossen, Germany). Velocity of impact was 5 m/s, and the duration of impact was 50 msec. The diameter of the impactor tip was 2.5 mm, and the depth of brain deformation was set at 3 mm. In sham-injured animals, the same surgical procedures were performed, but actual injury was not produced.

### Histology

At the end of behavioral testing (post-injury day 50), rats were weighed, anesthetized with pentobarbital (100 mg/kg), and transcardially perfused with phosphate-buffered saline followed by 10% formalin. Brains were removed, and lesions were visually confirmed. Brains were then serially sectioned in the coronal plane at 100-μm thickness using a vibrating microtome. Every fifth section was mounted on glass slides and stained using a standard thionin protocol. Neocortical volumes from ipsi- and contralateral hemispheres extending from ∼4.2 mm anterior to bregma (M1 and M2) and −6.0 mm posterior to bregma (V1B and V1M)^17^ were estimated using section tracings and the application of Cavalieri's unbiased estimator probe (MBF Bioscience, Williston, VT). This range encompassed the majority of the neocortex.

### Auditory testing apparatus

Auditory testing was performed in a treatment-blind manner 2 and 4 weeks post-TBI and involved a battery of progressively complex auditory discrimination tasks utilizing a modified acoustic startle paradigm. The task order was selected based on previous studies, which showed that step-wise exposure to progressively complex acoustic stimuli increases performance and the likelihood of deficit detection after brain injury.^7^ Modified acoustic startle paradigms (also referred to as pre-pulse inhibition) have been described extensively elsewhere.^7,18,19^ Here, subjects were placed on individual load cell platforms (Med Associates, Georgia, VT), which measured animals' ballistic motor response (in mV) to a startle eliciting stimulus (SES; 105-dB broadband white noise).

Depending on the task type (single-tone, silent-gap, or FM sweep), in a given trial, subjects were randomly presented with either cued or uncued stimuli before onset of the SES. Details are described for each task in the subsections below. Subjects were only presented with a single task type on a given day (Fig. 1). The maximum peak value (mV) defining the acoustic startle response (ASR) for each trial was isolated from a 200-ms window for each subject after the onset of each SES.^7,20^ The ASR represented one dependent variable in this paradigm. Attenuated response scores (ATT), a measure of cue discrimination, were calculated from the peak ASR (as measured by the load-cell displacement in mV), using the formula: (mean cued response/mean uncued response) × 100. Thus, ATT scores reflected a measurement of cue detection and were analyzed as a second dependent variable for all tasks.

### Single-tone tests

For both testing windows, the single-tone task was used to evaluate basic auditory acuity through pre-pulse inhibition before the assessment of more complex temporal processing (e.g., silent-gap and FM sweeps, similar to auditory cues used to evaluate humans).^14,19^ Single-tone sessions consisted of 104 trials (52 cued and 52 uncued) presented in a randomized fashion. Uncued trials included a background of silence followed by a 105-dB, 50-ms burst of white noise (SES). On cued trials, a 75-dB, 7-ms, 2300-Hz tone was presented 50 ms before the SES. Trial times (i.e., time between SES presentations) were randomized and varied in duration between 16 and 24 sec to eliminate the ability for subjects to habituate to the SES.

### Silent-gap tests

The silent-gap procedure is a common task used to assess basic auditory temporal processing in rodent models and has been used in humans for the same purpose.^7,19^ In each testing window, silent-gap testing began 1 day after the normal single tone task. Animals were given 1 day of testing within a series. Each session included 300 trials, each containing the randomized presentation of a variable duration silent-gap (0, 2, 5, 10, 20, 30, 40, 50, 75, or 100 ms) embedded in continuous 75-dB broadband white noise. Each silent-gap was presented 50 ms before a 105-dB burst of white noise (SES). Uncued trials utilized no gap presentation (0 ms) before the startle burst. The cue-burst interval was fixed at 50 ms.^7^

### Frequency-modulated sweep tests

The FM sweep task reflects the highest level of complexity in this study. Similar to the single-tone, each session consisted of 104 trials. Sessions involved a repeated presentation of background 75-dB FM sweeps ranging from 2300 to 1100 Hz of either 225- or 175-ms duration (two separate sessions). Each sweep was separated by an interval 200 ms longer than the sweep duration (either 225 or 175 ms) to maintain perceptual contiguity. On uncued trials, the last tail of the sweep was followed by 50 ms of silence, then by 105-dB, 50-ms SES. On cued trials, a reversal of the sweep occurred (low-high, 1100–2300 Hz), followed by 50 ms of silence, then the SES. These sessions were presented on the last 2 days of testing to maximize novel processing demand.

### Statistical analyses

Main effects were analyzed using repeated-measures analysis of variance (ANOVA) for session/day of testing, treatment, and cue (tone, gap, or FM sweep) for both ATT and absolute response (startle magnitude in mV). Simple-effects analyses were used when main effects or interactions were observed. For histological analysis, *t*-tests were used.

## Results

### Cortical volume reconstruction

**FIG. 2.**
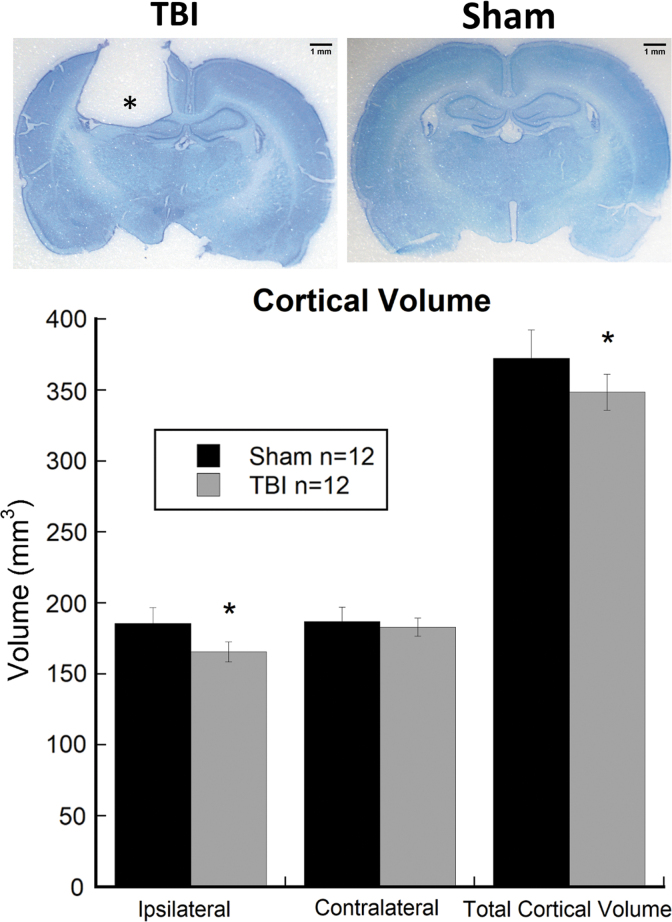
Histogram showing cortical volume measurements extending from ∼4.2 mm anterior to bregma (M1 and M2) and −6 mm posterior to bregma (V1B and V1M), as calculated by Cavalieri's estimator. Results show significant reductions in ipsilateral and total cortical volumes in TBI subjects (*n* = 12) as compared to shams (*n* = 12). *Indicates significant differences, *p* < 0.05. Volumes of contralateral cortices in TBI and sham-injured rats were statistically comparable. Data represent mean ± SEM. SEM, standard error of the mean; TBI, traumatic brain injury.

Cortical volume analysis from Cavalieri's estimation extended from ∼4.2 mm anterior to bregma (M1 and M2) to −6.0 mm posterior to bregma (V1B and V1M).^17^ Results from independent-samples *t*-tests showed significant effects of TBI for ipsilateral cortex (*t*_(22)_ = −5.223, *p* < 0.01) and total cortical volume (combined right and left; *t*_(22)_ = −3.514, *p* < 0.01), indicating that TBI subjects had significantly reduced cortical volumes as compared to shams (Fig. 2). Contralateral cortex alone was not significantly reduced in TBI subjects as compared to shams (*t*_(22)_ = −1.132, *p* > 0.05). All subjects in the TBI group had focal cortical lesions centered ∼3.0 mm posterior to bregma and 2.5 mm lateral to the midline. No such lesions were noted in brains of sham-injured rats (Fig. 2).

### Auditory processing

#### Single-tone

**FIG. 3.**
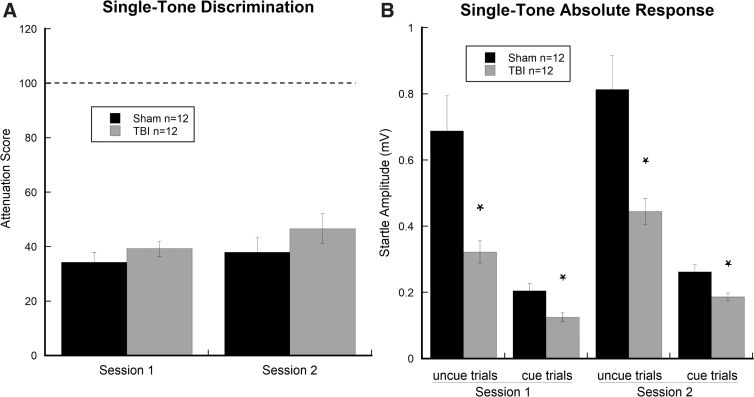
Graphs show (**A**) attenuation scores for sham and TBI subjects across the two single-tone testing sessions, indicating significant detection on the single-tone tasks for both groups (as measured by paired-samples *t*-tests mean cued and uncued trials within each group). (**B**) Absolute response scores (peek startle amplitude) for cued and uncued trials across the two testing sessions, indicating significantly reduced startle amplitudes in TBI subjects regardless of trial type or session as compared to sham subjects (*p < 0.05). Data are presented as mean ± SEM. Plot falling below dotted line indicates cue detection. SEM, standard error of the mean; TBI, traumatic brain injury.

Repeated-measures ANOVA for ATT showed no significant effects of Session (two levels) or Treatment (two levels; sham, *n* = 12; TBI, *n* = 12), indicating comparable single-tone performance by sham-injured versus TBI rats (Fig. 3A). Repeated-measures ANOVA for absolute startle amplitudes (as measured in mV) revealed a significant effect of Treatment (two levels; sham, *n* = 12; TBI, *n* = 12; *F*_(1, 22)_ = 13.018, *p* < 0.01), with TBI subjects showing significantly reduced startle amplitudes for both cued and uncued trials when compared to shams. Results also showed a significant effect of Cue (two levels; *F*_(1, 22)_ = 66.365, *p* < 0.001), indicating that startle responses were reduced for cued trials as compared to uncued trials regardless of the presence or not of TBI. For both sessions of the single-tone task, significant differences were observed between cued and uncued peak response scores for TBI rats (session one, *t*_(11)_ = 7.709, *p* < 0.001; session two, *t*_(11)_ = 6.401, *p* < 0.001) and shams (session one, *t*_(11)_ = 5.438, *p* < 0.001; session two, *t*_(11)_ = 6.194, *p* < 0.001; Fig. 3B). Results indicate that both groups significantly detected the single tone with a similar level of auditory acuity regardless of session of presentation or the presence of TBI, as indicated by significantly reduced startle amplitudes in cued (single-tone) when compared to uncued trials.

#### Silent-gap

**FIG. 4.**
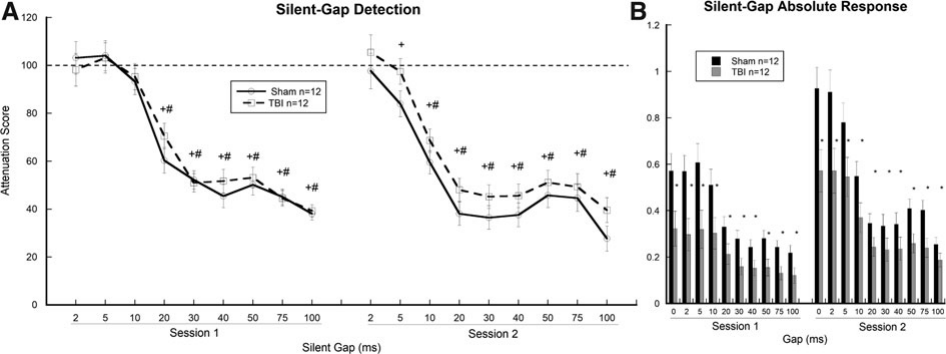
Graphs show (**A**) comparable basic auditory temporal processing of silent gaps in white noise for both groups in session one with both groups significantly discriminating 20- to 100-ms gaps. In contrast, session two revealed a shift in auditory acuity (experience effect), with sham subjects significantly detecting 5- to 100-ms gaps and TBI subjects having a detection range of 10–100 ms. ^+^Indicates a significant difference (*p* < 0.05) between average cued and uncued responses for sham rats; ^#^indicates a significant difference (*p* < 0.05) between average cued and uncued responses in TBI rats. (**B**) Absolute response scores for cued and uncued trials across the two testing sessions showing significantly reduced startle amplitudes in TBI subjects as compared to shams (*p < 0.05). Data represent mean ± SEM. Plot falling below dotted line indicates cue detection. SEM, standard error of the mean; TBI, traumatic brain injury.

Repeated-measures ANOVA for ATT scores across Session (two levels) × Gap (nine levels; 2, 5, 10, 20, 30, 40, 50, 75, and 100 ms) revealed a significant effect of Session (*F*_(1, 22)_ = 8.596, *p* < 0.01), indicating improved gap detection in session two as compared to session one. An effect of Gap (*F*_(1, 8)_ = 134.562, *p* < 0.001) was also observed, indicating improved gap detection for both groups as gap duration increased from 2 to 100 ms. A Session × Gap interaction was also observed (*F*_(8, 176)_ = 134.562, *p* < 0.001), reflecting greater improvements in gap detection for sham as compared to TBI subjects in session two (Fig. 4A). Based on this interaction, simple-effects analysis showed that sham-injured subjects had significantly better detection thresholds down to the 5-ms gap as compared to TBI subjects who were only able to significantly perceive down to a 10-ms gap (*p* < 0.01). A repeated-measures ANOVA for absolute startle amplitude (as measured in mV) revealed a significant effect of Treatment (two levels; sham, *n* = 12; TBI, *n* = 12; *F*_(1, 22)_ = 6.857, *p* < 0.05), with TBI subjects versus shams showing significantly reduced startle amplitudes for both sessions across trials regardless of cue. This finding parallels a similarly observed reduction in overall startle reactivity for TBI subjects in the single-tone task. In addition, a significant effect of Gap (*F*_(1, 22)_ = 25.681, *p* < 0.001) was observed, reflecting a decrease in overall startle magnitudes as gaps increased. Interestingly, a Gap × Treatment interaction (*F*_(1, 22)_ = 25.681, *p* < 0.001) was also observed, with sham subjects showing greater relative reductions in startle magnitude as compared to TBI subjects when gap duration increased from 0 (uncued) to 100 ms. Based on this interaction, simple-effects analysis showed that for both sessions of the silent-gap task, significant differences were observed between cued and uncued peak response scores for TBI rats (session one: 20, 30, 40, 50, 75, and 100 ms [*p* < 0.01]; session two: 10, 20, 30, 40, 50, 75, and 100 ms [*p* < 0.01]) and sham-injured subjects (session one: 20, 30, 40, 50, 75, and 100 ms [*p* < 0.01]; session two: 5, 10, 20, 30, 40, 50, 75, and 100 ms [*p* < 0.01]). These results support overall improvement in gap detection for sham as compared to TBI subjects across sessions (Fig. 4B).

### Frequency-modulated sweep

**FIG. 5.**
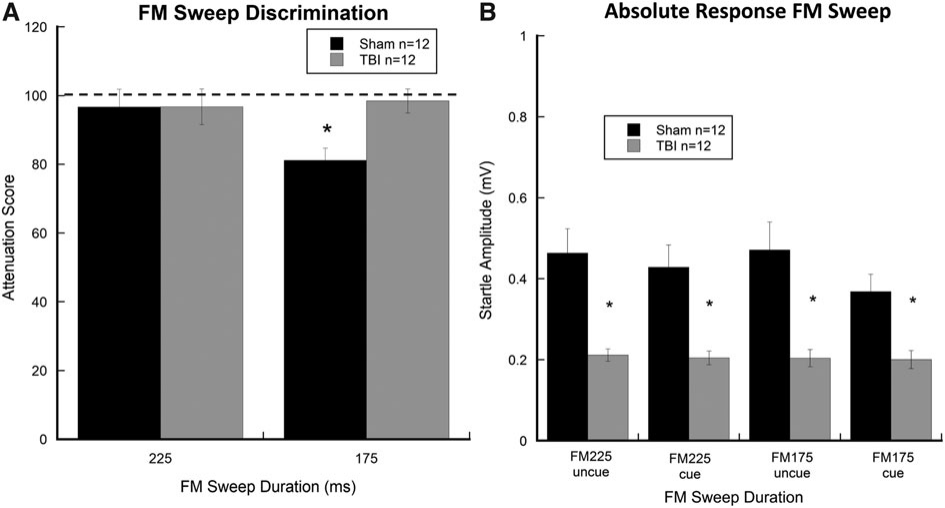
Graphs show (**A**) an interaction between FM sweep discrimination across interstimulus intervals (225 and 175 ms) as a function of injury, as measured by ATT scores for sham and TBI subjects, with sham subjects showing significant detection of the session two sweep (175 ms) as compared to TBI rats (*p < 0.05). (**B**) Absolute response scores (mV) are depicted for cued and uncued trials across the FM sweep-cue intervals (FM225 uncued, FM225 cued, FM175 uncued, and FM175 cued), showing significantly reduced startle amplitudes in TBI subjects regardless of cue presence or sweep interval (*p < 0.05). Only sham subjects showed attenuation of the startle response to cue presentation across FM sweep durations. Data are presented as mean ± SEM. Plot falling below dotted line indicates cue detection. ATT, attenuation; FM, frequency-modulated; SEM, standard error of the mean; TBI, traumatic brain injury.

For FM sweeps, repeated-measures ANOVA for ATT scores revealed a Sweep Duration (two levels; 225 and 175 ms) × Treatment (two levels; TBI, *n* = 12; sham, *n* = 12) interaction (*F*_(1, 22)_ = 4.884, *p* < 0.05). This result is indicative of improved sham performance on the 175-ms sweep, showing 20% startle attenuation as compared to TBI subjects (*p* < 0.01; Fig. 5A). A repeated-measures ANOVA for absolute startle amplitudes (as measured in mV) across both sweep durations (225 and 175 ms) revealed a significant effect of Treatment (*F*_(1, 22)_ = 22.704, *p* < 0.05), with TBI subjects (vs. shams) showing significantly reduced startle amplitudes for both sweep durations (Fig. 5B). This finding parallels the significant reduction in startle magnitude observed for both single-tone and silent-gap tasks in TBI subjects, further highlighting a global deficit in startle reactivity regardless of cue type. Further, main effects of Cue (*F*_(1, 22)_ = 7.717, *p* < 0.05) and a Cue × Treatment interaction (*F*_(1, 22)_ = 5.672, *p* < 0.05) were observed, indicating that startle reactivity was diminished in cued (sweep reversal) trials, and only for shams as compared to TBI rats (Fig. 5B).

## Discussion

TBI results in diffuse cortical and subcortical damage associated with cellular destruction, axon shearing, excitatory amino acid toxicity, and neuroinflammation, which often lead to substantial brain tissue loss and, consequently, cross-modal sensory and learning impairments.^3^ Consistent with previous findings in TBI rodent models, we report significant reductions in ipsilateral and total cortical volume in TBI subjects as compared to shams. In contrast, the contralateral hemisphere did not show any significant cortical loss in TBI subjects as compared to shams. In TBI patients and rodent models, the cellular and gross anatomical effects of injury are often accompanied by central auditory processing impairments and reduced startle reactivity.^8,12,21–23^ Importantly, patients with varying TBI severity show reductions in brainstem-mediated startle magnitude and deficits in auditory temporal processing.^25,28^ This body of research highlights a need to incorporate a range of auditory processing measures into the assessment of animal models of TBI for the development of more-effective intervention strategies.

Here, we evaluated the effects of TBI on startle reactivity and central auditory processing utilizing a modified acoustic startle paradigm encompassing a series of progressively complex cue burst combinations (single-tone, silent-gap, and FM sweep). Auditory tasks were selected to evaluate a range of temporal (timing) and spectral (frequency) processing abilities in addition to the role of cue type (single-tone, silent-gap, or FM sweep) on startle intensity. On the single-tone task, TBI and sham subjects showed comparable levels of discrimination as evidenced by reductions in startle amplitude for cued versus uncued trials (pre-pulse inhibition). Proficient single-tone cue detection is indicative of intact peripheral hearing in TBI and typical subjects.^7,8,21^ Importantly, TBI subjects showed significantly reduced ASR as compared to sham subjects, on both cued and uncued trials across both sessions. This finding replicates previous reports of impaired startle reactivity in rodent models of TBI in the presence of intact pre-pulse inhibition for pure tones and parallels the profile observed in TBI patients.^24,25^

Silent-gaps of variable duration embedded in broadband white noise have been used to assess auditory temporal acuity across a wide range of typical and pathological states in patients and rodent models.^18,26.27^ During session one of the silent-gap task, both groups showed significant detection of gaps ranging from 20 to 100 ms in duration, with neither group being able to detect 2- to 10-ms gaps. In contrast, session two showed a shift toward greater temporal acuity in both groups, with greater improvement noted in shams compared to TBI subjects. Specifically, shams were able to significantly detect silent-gaps ranging from 5 to 100 ms in duration, whereas TBI subjects were only able to discriminate 10- to 100-ms gaps. The presence of a Gap by Treatment interaction for absolute startle magnitude indicated that as gap duration decreased, startle intensity was more reduced in sham-injured as compared to TBI subjects. As observed in other neuropathology models, disparate shifts in acuity for short-duration gaps (5 and 10 ms) indicate temporal processing deficits.^14,18^ Interestingly, similar deficits in auditory temporal processing have been reported widely across the range of neurodevelopmental and acquired brain disorders.^12–14,23,28^

These deficits are most frequently observed when the rate of stimulus presentation decreases (e.g., from 100 to 2 ms in duration) and/or as task/cue complexity increases (e.g., as in the current study, single-tone, silent-gaps, and FM sweeps).^21,22,28^ In both cases, the relative perceptual processing demand increases. Importantly, presentation of a novel FM sweep task at the end of session two revealed a significant defect in processing the short-duration FM 175-ms cue in TBI subjects compared to shams. These findings parallel previous reports from models showing that repeated acoustic testing sessions, similar to those presented in the current study, improved auditory acuity in sham subjects when compared to those with traumatic injury or neocortical developmental malformations.^20,29^ Researchers have reported that auditory deficits in rodent models typically emerge after repeated testing sessions and relative increases in task demand (e.g., single-tone, silent-gaps, two-tone, and FM sweep).^7,8,20,29^ The current findings provide support for the notion that auditory temporal processing may be a core deficit in TBI. Given the present findings, assessment of auditory sensory processing should be undertaken when investigating intervention or rehabilitative approaches for TBI.

In addition to observed impairments in rapid-gap and FM-sweep discrimination, TBI subjects showed nearly universal reductions in absolute startle magnitude across cued and uncued presentations of the single-tone, silent-gap, and FM-sweep tasks (see Figs. 3B, 4B, and 5B). Previous reports show significant reductions in startle magnitude, regardless of injury severity, in both TBI patients and animal models of TBI.^24,30,31^ Interestingly, reduced ASR in TBI appears to be independent of auditory acuity given that basic pre-pulse inhibition of the ASR remains intact, as evidenced in the current study by comparable single-tone processing in both groups. Importantly, using a fluid percussion model in rats, Sinha and colleagues^30^ reported that mild TBI produced a progressive upregulation of proinflammatory cytokines in the caudal pontine reticular nucleus (PnC), a key structure involved in the startle response. This finding suggests that global inflammatory responses distal to the original site of injury may play a role in modulating startle reactivity.

Presently, ASR attenuation was comparable between sham and TBI subjects for the single-tone task despite TBI subjects showing nearly half the startle magnitude of their sham counterparts (see Fig. 3B). Whereas short-latency ASR is mediated by well-mapped brain stem synapses, attenuation of the startle response may involve descending input to the PnC from inferior and superior colliculi, thalamic, and cortical circuits.^7^ Thus, disruption of higher-order processing centers and descending pathways, as occurs in TBI, may contribute to alterations in the complex auditory processing observed in the present study, independent of PnC regulation of ASR.^7,30,32,33^ Given the high personal, social, and economic cost of TBI, the widespread adoption of these auditory tasks may help to improve the replicability and throughput of pre-clinical translational studies.

The limitation of the present study is the focus on male rats. Increasing evidence indicates that sex and circulating levels of female sex hormones (the phase of the estrous cycle during which injury occurred) may affect neurological outcome after TBI in both laboratory animals and humans.^34–37^ Sex hormones may also have an effect on post-traumatic brain inflammatory response,^38^ which, as discussed above, could contribute to behavioral deficits observed after TBI. Although the findings of this study may not entirely apply to female animals, they represent an important stepping stone for further mixed-sex investigations of deficits in the processing of auditory information occurring after TBI.

## Abbreviations Used

ANOVA: analysis of variance
ASR: acoustic startle response
ATT: attenuated response scores
CCI: controlled cortical impact
FM: frequency-modulated
IACUC: Institutional Animal Care and Use Committee
PnC: caudal pontine reticular nucleus
RIH: Rhode Island Hospital
SES: startle eliciting stimulus
TBI: traumatic brain injury
